# Sitagliptin as a therapeutic approach for social anxiety disorder: the role of DPP4 and NPY in modulating social fear and comorbid depressive-like behavior in mice

**DOI:** 10.1038/s41386-025-02146-8

**Published:** 2025-06-09

**Authors:** Iulia Zoicas, Christiane Mühle, Stephan von Hörsten, Anne-Christine Plank, Johannes Kornhuber

**Affiliations:** 1 Department of Psychiatry and Psychotherapy, Friedrich-Alexander-Universität Erlangen-Nürnberg (FAU) and Universitätsklinikum Erlangen, Schwabachanlage 6, 91054 Erlangen, Germany; 2 Department of Experimental Therapy, Preclinical Experimental Center, Friedrich-Alexander-Universität Erlangen-Nürnberg (FAU) and Universitätsklinikum Erlangen, Palmsanlage 5, 91054 Erlangen, Germany; 3 Present Address: Department of Child and Adolescent Mental Health, Friedrich-Alexander-Universität Erlangen-Nürnberg (FAU) and Universitätsklinikum Erlangen, Schwabachanlage 6, 91054 Erlangen, Germany

**Keywords:** Emotion, Fear conditioning

## Abstract

We have previously shown that neuropeptide Y (NPY) reduces social fear in an animal model that closely mimics the key behavioral symptoms of social anxiety disorder (SAD). Since NPY cannot yet be routinely administered to patients, we investigated the effects of sitagliptin, a dipeptidyl peptidase-4 (DPP4) inhibitor approved for the treatment of type 2 diabetes mellitus, on social fear and comorbid depression in mice. In addition to its well-known effects on glucose metabolism, sitagliptin also prevents the degradation of NPY, thereby increasing its concentration in the blood and the brain. We show that sitagliptin administration via drinking water (50 and 100 mg/kg/day, for 4 weeks) not only reduced social fear but also prevented the onset of comorbid depressive-like behavior in outbred CD1 mice. A similar phenotype was observed in homozygous DPP4-deficient mice, emphasizing the role of DPP4 in regulating these behaviors. However, in NPY-deficient mice, sitagliptin showed reduced efficacy, suggesting that NPY plays an important role in mediating the effects of sitagliptin on social fear and comorbid depression. These findings have important clinical implications, indicating that early intervention with sitagliptin could be an effective strategy for treating SAD, alleviating both core symptoms and reducing the risk of developing comorbid mood disorders that often complicate treatment outcomes.

## Introduction

Social anxiety disorder (SAD) is the second most common anxiety disorder, with a lifetime prevalence of around 12%, and is characterized by persistent fear and avoidance of social situations [1]. SAD is highly comorbid with major depressive disorder (affecting 35–70% of SAD patients), specific phobia (14–61%), panic disorder (5–27%), agoraphobia (8–45%), generalized anxiety disorder (0.6–27%), alcohol use disorders (up to 50%), obsessive compulsive disorder (2–19%) and posttraumatic stress disorder (3–16%) [1]. Except for specific phobia, SAD has an earlier onset, suggesting that SAD is a risk factor for developing additional psychiatric disorders [1]. Early diagnosis and intervention could help reduce this risk.

Animal models are essential for understanding the neurobiology of SAD and its comorbidities. They should ideally replicate both specific social anxiety (i.e., without any comorbidities) and social anxiety with comorbid conditions to provide valuable insights for developing targeted treatments for SAD patients with and without comorbidities. One such model is social fear conditioning (SFC), which mimics the key behavioral symptoms of SAD, like social avoidance and reduced social investigation [2, 3]. The SFC model has face, predictive and construct validity for human SAD [2, 4] and induces specific social fear without symptoms of generalized anxiety or depressive-like behavior for up to 2–3 weeks post-conditioning [2, 5]. A depressive-like phenotype can emerge later, around 5–6 weeks post-conditioning [5].

In previous studies, we demonstrated that central administration of neuropeptide Y (NPY), a neuropeptide well known for its fear-reducing properties in animal models of conditioned fear [6], effectively reduced SFC-induced social fear in male mice [7–9]. Central NPY also alleviated an antidepressant-resistant type of social fear in both male and female mice [10], supporting its potential in treating SAD. Elevated NPY levels have also been associated with reduced anxiety- and depressive-like behaviors, while lower NPY levels often correlate with heightened stress sensitivity and more pronounced depressive-like symptoms [11, 12]. This ability of NPY to modulate the stress response and to reduce both fear-related behaviors and anxiety-/depressive-like symptoms suggests that NPY may be a promising target for developing new therapies for SAD with comorbid depression. However, NPY has not yet been developed or approved for routine clinical use, and its administration to patients is currently restricted to controlled research settings.

One effective way to increase NPY concentration is by inhibiting its breakdown. Dipeptidyl peptidase-4 (DPP4) is an enzyme that cleaves the amino-terminal dipeptide from several peptides, including the incretin hormones glucagon-like peptide-1 (GLP-1) and glucose-dependent insulinotropic peptide (GIP), as well as other peptides such as NPY [13–16]. DPP4 inhibitors, also known as gliptins, are used in the treatment of type 2 diabetes mellitus to improve glucose homeostasis. They inhibit the degradation of GLP-1 and GIP, leading to an increased insulin secretion and reduced glucagon secretion [14, 15]. In addition to their effects on glucose metabolism, DPP4 inhibitors such as sitagliptin, vildagliptin, linagliptin and saxagliptin also inhibit the degradation of NPY, thereby increasing its blood concentration in healthy individuals and patients with type 2 diabetes mellitus [17–21]. In rodents, sitagliptin has been shown to increase NPY levels in brain regions such as the hippocampus, amygdala and hypothalamus [22]. Preclinical studies suggest that DPP4 inhibition may offer potential benefits for treating conditioned fear, anxiety and mood disorders. For example, sitagliptin exerts antidepressant-like effects in naïve mice [23]. DPP4 inhibitors also improve social interaction deficits [24] and reduce depressive-like behaviors in various animal models of depression, including chronic restraint stress [24], chronic unpredictable mild stress [25], high fat diet [24], diabetes [26] and morphine withdrawal [27]. Additional insights come from studies on DPP4-deficient rodents, which show enhanced extinction of cued fear [28], reduced anxiety-like behavior [29, but see 30] and reduced depressive-like behavior [31]. These DPP4-deficient animals also exhibit increased NPY levels in the cerebrospinal fluid [28], supporting the idea that the effects of DPP4 inhibition may be mediated, at least in part, by increased NPY levels.

In this study, we investigated whether early intervention with sitagliptin reduces SFC-induced social fear and prevents the development of comorbid depressive-like behavior. Additionally, we examined whether DPP4-deficient mice show reduced social fear compared with wild-type littermates, and whether NPY-deficient mice exhibit a diminished response to sitagliptin.

## Materials and methods

### Animals

Experiments were performed in male CD1 mice (Charles River, Sulzfeld, Germany), male and female homozygous DPP4-deficient (DPP4 −/−) [32] and NPY-deficient (NPY −/−) mice [33–35] (Supplementary Materials and Methods), as well as in wild-type controls (DPP4 + /+ and NPY + /+ mice), at an age of 8 − 9 weeks (see Table S1 for a detailed depiction of the experimental groups). Mice were individually housed for one week before experiments started and remained single-housed throughout the experiments. Age-, sex- and strain-matched wild-type mice were used as social stimuli for the SFC experiments. Mice were kept under standard laboratory conditions (12:12 light/dark cycle, lights on at 07:00 h, 22 °C, 60% humidity, food and water ad libitum). Experiments were performed during the light phase between 09:00 and 14:00 in accordance with the EU Directive 2010/63 for the protection of animals used for scientific purposes and were approved by the local government commission for animal protection (Regierung von Unterfranken).

### Gliptin treatment

Sitagliptin (sitagliptin phosphate; PHR1857, Sigma-Aldrich, Darmstadt, Germany) was administered for 4 weeks via drinking water at a daily dose of 50 mg/kg or 100 mg/kg [36, 37]. The treatment was started one day after SFC to avoid potential treatment-induced alterations in social fear memory consolidation and was maintained throughout the experiment. The mice’s body weight and drinking volume were assessed every two to three days, and the sitagliptin solution was freshly prepared accordingly.

### Social fear conditioning (SFC) paradigm

To induce social fear, mice were conditioned during SFC on day 1, and social investigation was assessed during social fear extinction on day 30 as a read-out of social fear.

#### SFC (day 1)

SFC was performed with a computerized fear conditioning system (TSE System GmbH, Bad Homburg, Germany), as previously described [3, 4, 7–10, 38–40]. Mice were placed in the conditioning chamber (45 × 22 × 40 cm) and, after a 30-sec habituation, an empty wire mesh cage (7 × 7 × 6 cm) was placed as a non-social stimulus near one of the short walls. After 3 min, the non-social stimulus was replaced by an identical cage containing an unfamiliar age-, sex- and strain-matched mouse. Unconditioned control mice (SFC –) were allowed to investigate this social stimulus for 3 min, whereas socially fear conditioned mice (SFC + ) received a 1-sec mild electric foot-shock (0.7 mA) each time they investigated, i.e., made direct contact with the social stimulus. Mice received between one and four foot-shocks with a variable inter-shock interval, depending on when direct social contact was made. The number of foot-shocks was assessed as a measure of distress and of social fear learning. Mice were returned to their home cage when no further social contact was made for 2 min (average duration of SFC approximately 10 min). The time spent investigating the non-social stimulus was analyzed as a preconditioning measure of non-social anxiety.

#### Social fear extinction (day 30)

After 4 weeks of sitagliptin treatment, mice were exposed in their home cage to three non-social stimuli, to assess non-social investigation as a parameter of non-social fear. Mice were then exposed to six unfamiliar social stimuli to assess social investigation as a parameter of social fear. Each stimulus was presented for 3 min, with a 3-min inter-exposure interval. The test was recorded and analyzed using JWatcher (V 1.0, Macquarie University, Sydney, Australia, and UCLA, Los Angeles, CA, USA). Non-social investigation was defined as direct sniffing of the empty cage, whereas social investigation was defined as direct sniffing of the cage and/or the social stimulus inside the cage.

### Elevated plus-maze (EPM) test

To investigate whether SFC leads to the development of a comorbid anxious phenotype, mice were tested in the EPM on day 34 as previously described [5, 41, 42]. The test was recorded and analyzed using JWatcher. The time spent on the open arms (150 lx) indicated non-social anxiety-like behavior. The number of entries into the closed arms (30 lx) during the 5-min testing period indicated locomotor activity.

### Forced swim test (FST)

To investigate whether SFC leads to the development of a comorbid depressive-like phenotype, mice were tested in the FST on day 39 as previously described [5, 41, 42]. Mice were individually placed into a Plexiglas cylinder filled with 25 °C water for 6 min. The test was recorded and analyzed using JWatcher. An increased immobility time during the last 4 min of the test indicated a depressive-like phenotype.

### Quantification of DPP4 activity, DPP4 protein levels and baseline corticosterone (CORT) levels in the serum

To verify whether the selected doses of sitagliptin effectively inhibited DPP4 activity, CD1 mice were rapidly killed one day after behavioral testing, on day 40. Trunk blood was collected via cardiac puncture and left to coagulate. After centrifugation (4 °C, 4000 rpm, 10 min), the serum was extracted and stored at –80 °C.

DPP4 activity was quantified in 4 µl serum duplicates using the DPP4 Activity Assay Kit (MAK088, Sigma-Aldrich, Saint Louis, MO, USA), based on the cleavage of the non-fluorescent substrate H-Gly-Pro-AMC to release the fluorescent product7-Amino-4-Methyl Coumarin (AMC). The kinetics of fluorescence increase at 37 °C were assessed on a CLARIOstar Plus multi-mode plate reader (BMG LABTECh GmbH, Ortenberg, Germany) and corrected for the AMC release in a reaction with sitagliptin as DPP4 inhibitor. All samples were assessed within one assay with an intra-assay coefficient of variation (CV) of 2%.

DPP4 protein levels were quantified in 1 µL serum duplicates using the Mouse DPPIV ELISA Kit (RAB0148, Sigma-Aldrich, Saint Louis, MO, USA), based on a standard curve ranging from 0.3 to 100 ng/mL, within one assay with an intra-assay CV of 3%.

To assess whether SFC and/or sitagliptin treatment affected baseline CORT levels, CORT was quantified in 4 µL serum duplicates using a competitive CORT ELISA Kit (EIA-4164, DRG Instruments GmbH, Marburg, Germany), based on a standard curve ranging from 5 to 240 nmol/L. The intra-assay CV was 5% and the inter-assay CV was 12%.

### Statistical analysis

For statistical analysis, IBM SPSS (Version 28.0, SPSS Inc., Chicago, IL, USA) was used. Data were analyzed by Student’s t-test, one-way, two-way or three-way ANOVA for repeated measures, followed by Bonferroni’s post hoc analysis whenever appropriate. Statistical significance was set at *p* < 0.05.

## Results

Sitagliptin did not influence body weight in either CD1 mice (Fig. S1; Table S2) or NPY-deficient mice (Fig. S2; Table S2). Sitagliptin consumption was consistent within each group, and neither sitagliptin nor its taste influenced fluid intake (Fig. S1; Fig. S2; Table S2). Male and female mice were pooled together, as no sex differences in behavior were observed (Table S3). Sex differences in body weight and fluid intake in NPY-deficient mice are provided in the supplementary information (Fig. S2; Table S2).

### Sitagliptin reduces social fear and prevents the development of comorbid depressive-like behavior in CD1 mice

**Fig. 1:**
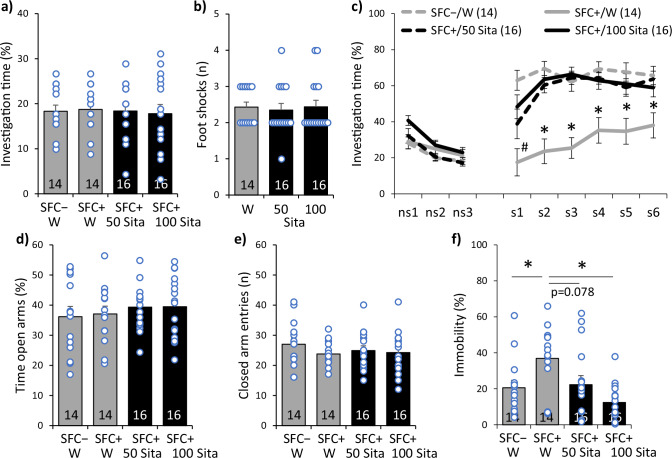
Sitagliptin reduces social fear and prevents the development of comorbid depressive-like behavior in male CD1 mice. **a** Preconditioning investigation of the non-social stimulus (empty cage) shown by unconditioned (SFC − ) and conditioned (SFC + ) mice during social fear conditioning (SFC) on day 1. **b** Number of foot-shocks received during SFC. **c** Investigation of the non-social (ns1 − ns3) and social (cages with mice; s1 − s6) stimuli during social fear extinction on day 30; # *p* < 0.05 versus SFC − /W and SFC + /100 Sita; * *p* < 0.05 versus all groups. **d** Time spent on the open arms of the elevated plus-maze (EPM) and **e** number of closed-arm entries on day 34. **f** Immobility time in the forced swim test (FST) on day 39; * *p* < 0.05. Mice were administered sitagliptin (Sita; 50 or 100 mg/kg/day) via drinking water (W). Data represent means ± SEM and numbers on the bars and in parentheses indicate group sizes.

During SFC on day 1, all groups of mice showed similar investigation of the non-social stimulus (empty cage), reflecting similar preconditioning non-social anxiety (Fig. 1a; F(3,56) = 0.058; *p* = 0.981). All SFC + mice received a similar number of foot-shocks, reflecting similar levels of distress during conditioning and similar social fear learning between the groups (Fig. 1b; F(2,43) = 0.092; *p* = 0.912). During social fear extinction on day 30, all mice showed similar non-social investigation (i.e., investigation of the three empty cages), indicating that SFC did not induce an unspecific non-social fear and that sitagliptin did not alter non-social investigation (Fig. 1c). Water-drinking SFC + mice showed reduced social investigation (i.e., investigation of the six social stimuli) compared with all other groups, reflecting social fear (Fig. 1c; group effect F(3,56) = 17.494; *p* < 0.001; stimulus x group effect F(24,448) = 6.603; *p* < 0.001). Both doses of sitagliptin, however, increased social investigation to levels observed in SFC– mice starting from the first (100 mg/kg/day) and second (50 mg/kg/day) social stimulus, reflecting significantly reduced social fear in SFC + mice after sitagliptin treatment.

On day 34, all mice showed similar non-social anxiety-like behavior (Fig. 1d; F(3,56) = 0.395; *p* = 0.757) and similar locomotor activity (Fig. 1e; F(3,56) = 0.672; *p* = 0.573) on the EPM, as reflected by similar time spent on the open arms and similar number of entries into the closed arms between the groups, respectively. However, on day 39, water-drinking SFC + mice showed increased immobility in the FST compared with water-drinking SFC − mice (Fig. 1f; F(3,56) = 5.550; *p* = 0.002), indicating that SFC induced a depressive-like phenotype. This depressive-like phenotype was not observed in sitagliptin-treated mice, indicating that sitagliptin prevented the development of comorbid depressive-like behavior in SFC + mice.

**Fig. 2:**
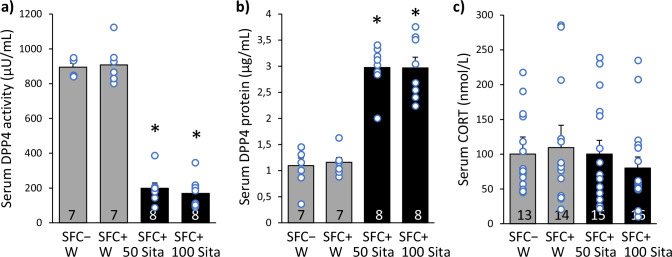
Sitagliptin reduces DPP4 activity, increases DPP4 protein levels and does not alter baseline corticosterone (CORT) levels in male CD1 mice. Mice were administered sitagliptin (Sita; 50 or 100 mg/kg/day) via drinking water (W). **a** Dipeptidyl dipeptidase 4 (DPP4) activity, **b** DPP4 protein levels and **c** baseline CORT levels in serum after 39 days of sitagliptin treatment. Data represent means + SEM and numbers on the bars indicate group sizes. * *p* < 0.05 versus SFC − /W and SFC + /W.

Sitagliptin treatment effectively reduced DPP4 activity (Fig. 2a; F(3,26) = 65.249; *p* < 0.001) and increased DPP4 protein levels (Fig. 2b; F(3,26) = 44.675; *p* < 0.001) in the serum, regardless of the dose applied, supporting previous findings in both blood and brain [43–45]. This indicates that the selected sitagliptin doses were appropriate for the intended therapeutic effect. Neither SFC nor sitagliptin treatment altered the baseline serum CORT levels (Fig. 2c; F(3,54) = 0.299; *p* = 0.826), suggesting that the observed effects on behavior were not mediated by changes in baseline stress levels. In a pilot analysis, sitagliptin treatment (50 mg/kg/day) increased serum NPY protein levels in socially fear conditioned male CD1 mice (T(11) = −2.349; *p* = 0.039; Fig. S3), confirming previous findings [17, 18].

### DPP4 deficiency reduces social fear and prevents the development of comorbid depressive-like behavior

**Fig. 3:**
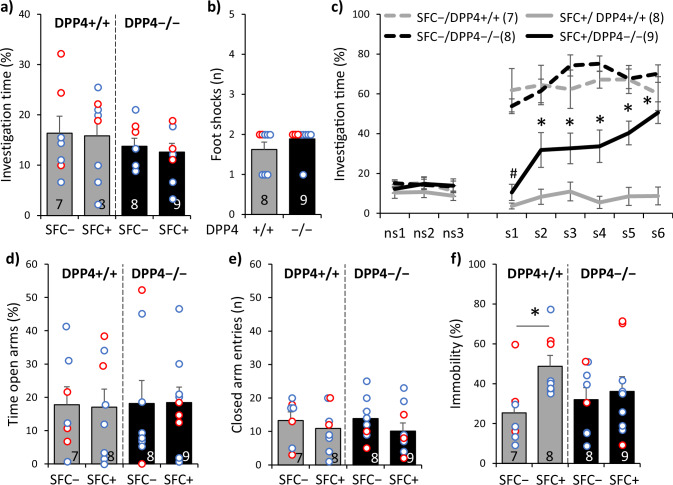
DPP4 deficiency reduces social fear and prevents the development of comorbid depressive-like behavior in male and female mice. **a** Preconditioning investigation of the non-social stimulus (empty cage) shown by unconditioned (SFC − ) and conditioned (SFC + ) homozygous dipeptidyl dipeptidase 4-deficient mice (DPP4 − /−) and wild-type controls (DPP4 + /+) during social fear conditioning (SFC) on day 1. **b** Number of foot-shocks received during SFC. **c** Investigation of the non-social (ns1 − ns3) and social (cages with mice; s1 − s6) stimuli during social fear extinction on day 30; # *p* < 0.05 SFC + versus SFC − , independent of genotype; * *p* < 0.05 versus SFC − /DPP4 − /− and SFC + /DPP4 + /+. **d** Time spent on the open arms of the elevated plus-maze (EPM) and **e** number of closed-arm entries on day 34. **f** Immobility time in the forced swim test (FST) on day 39; * *p* < 0.05. Data represent means ± SEM and numbers on the bars and in parentheses indicate group sizes. Blue and red dots represent individual male and female mice, respectively.

During SFC on day 1, all mice showed similar preconditioning non-social anxiety (Fig. 3a; F(1,28) = 0.017; *p* = 0.897). All SFC + mice received a similar number of foot-shocks (Fig. 3b; T(15) = − 1.265; *p* = 0.225). During social fear extinction on day 30, all mice showed similar non-social investigation (Fig. 3c). Both SFC + /DPP4 −/− and SFC + /DPP4 + /+ mice showed reduced social investigation compared with respective SFC − mice, reflecting social fear (Fig. 3c; conditioning x genotype effect F(1,28) = 6.728; *p* = 0.015; stimulus x genotype effect F(8,224) = 4.090; *p* < 0.001). SFC + /DPP4 −/− mice, however, showed higher social investigation compared with SFC + /DPP4 + /+ mice starting from the second social stimulus, reflecting reduced social fear.

On day 34, all mice showed similar non-social anxiety-like behavior (Fig. 3d; F(1,28) = 0.008; *p* = 0.931) and similar locomotor activity (Fig. 3e; F(1,28) = 0.078; *p* = 0.783) on the EPM. However, on day 39, SFC + /DPP4 + /+ but not SFC + /DPP4 −/− mice showed increased immobility in the FST compared with respective SFC − mice (Fig. 3f; conditioning effect F(1,28) = 4.450; *p* = 0.044; conditioning x genotype effect F(1,28) = 2.177; *p* = 0.151), indicating that DPP4 deficiency prevented the development of comorbid depressive-like behavior in SFC + mice.

### Sitagliptin reduces social fear and prevents the development of comorbid depressive-like behavior in NPY + /+ mice; reduced efficacy in NPY −/− mice

**Fig. 4:**
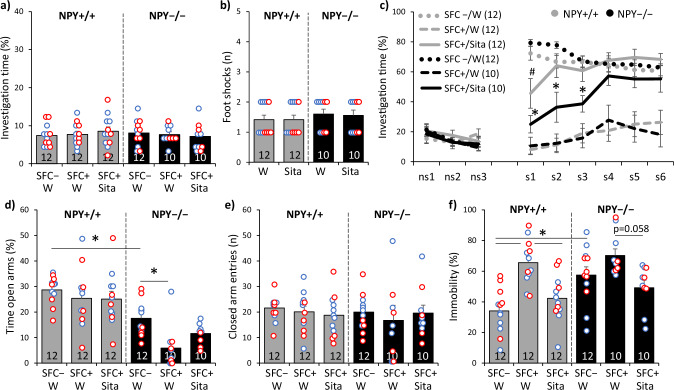
Sitagliptin reduces social fear and prevents the development of comorbid depressive-like behavior in NPY + /+ mice; reduced efficacy in NPY − /− mice. **a** Preconditioning investigation of the non-social stimulus (empty cage) shown by unconditioned (SFC − ) and conditioned (SFC + ) homozygous neuropeptide Y-deficient mice (NPY − /−) and wild-type controls (NPY + /+) during social fear conditioning (SFC) on day 1. **b** Number of foot-shocks received during SFC. **c** Investigation of the non-social (ns1 − ns3) and social (cages with mice; s1 − s6) stimuli during social fear extinction on day 30; # *p* < 0.05 versus SFC − /W/NPY + /+ and SFC + /W/NPY + /+; * *p* < 0.05 versus SFC − /W/NPY − /−. **d** Time spent on the open arms of the elevated plus-maze (EPM) and **e** number of closed-arm entries on day 34. **f** Immobility time in the forced swim test (FST) on day 39; * *p* < 0.05. Mice were administered sitagliptin (Sita; 100 mg/kg/day) via drinking water (W). Data represent means ± SEM and numbers on the bars and in parentheses indicate group sizes. Blue and red dots represent individual male and female mice, respectively.

During SFC on day 1, all mice showed similar preconditioning non-social anxiety (Fig. 4a; F(5,62) = 0.326; *p* = 0.895). All SFC + mice received a similar number of foot-shocks (Fig. 4b; F(3,40) = 0.303; *p* = 0.823). During social fear extinction on day 30, all mice showed similar non-social investigation (Fig. 4c). Both water-drinking SFC + /NPY −/− and SFC + /NPY + /+ mice showed reduced social investigation compared with respective water-drinking SFC − mice, reflecting social fear (Fig. 4c; group effect F(5,62) = 25.368; *p* < 0.001; stimulus x group effect F(40,496) = 8.874; *p* < 0.001). In SFC + /NPY + /+ mice, sitagliptin (100 mg/kg/day) increased social investigation to levels observed in SFC – /NPY + /+ mice starting from the second social stimulus, reflecting significantly reduced social fear. In SFC + /NPY −/− mice, however, sitagliptin showed a reduced efficacy as it increased social investigation to levels observed in SFC – /NPY −/− mice starting from the fourth social stimulus.

On day 34, NPY + /+ mice showed similar non-social anxiety-like behavior in the EPM. SFC – /NPY −/− mice, however, were more anxious than SFC – /NPY + /+ mice, reflecting elevated baseline anxiety levels in NPY −/− mice (Fig. 4d; F(5,62) = 12.731; *p* < 0.001). Interestingly, SFC further increased anxiety levels in NPY −/− mice, although such an effect was not observed in NPY + /+ mice nor in CD1 or DPP4 + /+ mice. This indicates that SFC induces a comorbid anxious phenotype specifically in individuals with increased baseline anxiety levels. Sitagliptin treatment attenuated, but did not reverse the SFC-induced anxious phenotype in SFC + /NPY −/− mice (Fig. 4d; *p* = 0.128). The locomotor activity on the EPM was similar between the groups (Fig. 4e; F(5,62) = 0.285; *p* = 0.919).

On day 39, water-drinking SFC + /NPY + /+ mice showed increased immobility in the FST compared with water-drinking SFC −/NPY + /+ mice (Fig. 4f; F(5,62) = 9.375; *p* < 0.001). This depressive-like phenotype was not observed in sitagliptin-treated NPY + /+ mice, indicating that sitagliptin prevented the development of comorbid depressive-like behavior in wild-type SFC + mice. SFC – /NPY −/− mice showed increased immobility compared with SFC – /NPY + /+ mice, reflecting elevated baseline levels of depressive-like behavior in NPY −/− mice (Fig. 4f; *p* = 0.006), and SFC did not further accentuate this depressive-like phenotype in NPY −/− mice. Sitagliptin treatment, however, tended to attenuate the depressive-like behavior in SFC + /NPY −/− mice (Fig. 4f; *p* = 0.058).

## Discussion

We demonstrate that the DPP4 inhibitor sitagliptin significantly reduces social fear in an animal model that closely mimics the key behavioral symptoms of SAD, while also preventing the onset of comorbid depressive-like behavior in CD1 mice. Similarly, homozygous DPP4-deficient mice displayed reduced social fear and did not develop comorbid depressive-like behavior, mirroring the effects of sitagliptin treatment. A reduced efficacy of sitagliptin was observed in homozygous NPY-deficient mice, suggesting that NPY plays an important role in mediating sitagliptin’s effects on social fear and depressive-like behavior. These findings provide new insights into the therapeutic potential of early intervention with sitagliptin for SAD, not only in alleviating symptoms but also in reducing the risk of comorbid mood disorders.

Our findings contribute to the growing body of evidence supporting the therapeutic potential of DPP4 inhibition in alleviating symptoms of anxiety and mood disorders. Previous studies have shown that DPP4 inhibitors improve social interaction deficits [24] and reduce depressive-like behaviors in various animal models of depression, including chronic restraint stress [24], chronic unpredictable mild stress [25], high fat diet [24], diabetes [26] and morphine withdrawal [27]. Our study extends these observations by showing that early treatment with sitagliptin has the potential not only to reduce social fear but also to prevent the onset of comorbid anxiety- and depressive-like behavior. This is particularly relevant for SAD, as comorbid anxiety and mood disorders are common and often complicate treatment outcomes [1]. While a larger sample size would be needed for more conclusive results, our findings suggest that both males and females could benefit from sitagliptin treatment.

The observation that DPP4-deficient mice display reduced social fear and do not develop comorbid depressive-like behavior further emphasizes the importance of DPP4 in regulating these behaviors. Notably, the fear learning processes and the initial expression of social fear in response to the first social stimulus were not altered in DPP4-deficient mice, ruling out potential impairments in fear memory. Instead, DPP4-deficient mice displayed a facilitated extinction of social fear, supporting previous reports of enhanced cued fear extinction in DPP4-deficient rats [28]. As fear extinction is a form of associative learning, this finding might suggest that DPP4 deficiency positively influences cognitive function. Supporting this hypothesis, DPP4 inhibition reduced cognitive impairments associated with neurodegenerative [46–48] and metabolic disorders [36, 49]. Additionally, DPP4-deficient rats show unaltered pain perception [28, 50], suggesting that the facilitated extinction of cued and social fear is not due to changes in pain sensitivity.

The reduced efficacy of sitagliptin observed in homozygous NPY-deficient mice indicates that NPY plays an important role in mediating sitagliptin’s effects on social fear and comorbid depressive-like behavior. Sitagliptin inhibits the degradation of NPY, resulting in elevated NPY levels in the blood [17, 18] (Fig. S3) and in brain regions such as the hippocampus, amygdala and hypothalamus [22]. Elevated NPY levels have been shown to reduce social fear in our experimental paradigm [7–10], impair the retention and retrieval of cued fear [51, 52] and facilitate the extinction of both cued and contextual fear [52–54]. Additionally, both sitagliptin and NPY have been linked to increased levels of brain-derived neurotrophic factor (BDNF) [46, 48, 55], a key molecule for neuronal survival, growth, differentiation and synaptic plasticity. BDNF has been shown to reduce conditioned fear and prevent the onset of stress-induced depressive-like behaviors [56–58]. Moreover, sitagliptin, NPY and BDNF are all implicated in promoting hippocampal neurogenesis, which is essential for memory formation, fear extinction and the mitigation of depressive-like behavior [59–61]. Although sitagliptin elevated blood NPY levels in socially fear-conditioned male CD1 mice (Fig. S3) and NPY is known to efficiently cross the blood-brain barrier [62], it remains unclear whether sitagliptin also increases brain NPY levels in these mice. The absence of this data represents a limitation of the study and warrants further investigation. The reduced efficacy, rather than a complete lack of effect, of sitagliptin in homozygous NPY-deficient mice also suggests that while NPY plays an important role, it is unlikely the sole mediator of sitagliptin’s effects. GLP-1 and GIP, other molecules whose degradation is inhibited by sitagliptin, may also contribute to the observed effects. GLP-1 analogs have been shown to alleviate social interaction deficits and reduce anxiety- and depressive-like behaviors in animal models of depression [63, 64] and diabetes [65, 66]. Notably, GLP-1 and GIP also promote synaptic plasticity, stimulate neurogenesis and promote neuroprotection [63, 64, 67, 68]. Importantly, DPP4-deficient mice and rats exhibit elevated levels of GLP-1, NPY and BDNF [28, 69], which are likely key factors contributing to their phenotype observed in this study. Thus, sitagliptin might reduce social fear and prevent the onset of comorbid depressive-like behavior through multiple interconnected pathways, which will be investigated in further studies.

Interestingly, while no effects of SFC on non-social anxiety-like behavior were observed in CD1 [5], C57Bl/6 [5] or DPP4-deficient mice, SFC did induce an anxious phenotype in NPY-deficient mice. Since NPY-deficient mice exhibited heightened anxiety levels compared with wild-type controls, as reported in previous studies [34, 70], it appears that SFC exacerbates anxiety in rodents with pre-existing heightened anxiety traits. Interestingly, sitagliptin has been shown to exert anxiolytic-like effects in rodents with elevated anxiety levels [48], but not in those without such heightened anxiety [23]. Overall, these findings suggest that both the comorbid anxiety induced by SFC and the efficacy of sitagliptin in modulating anxiety-like behavior may be influenced by baseline anxiety levels.

In conclusion, our findings demonstrate that both sitagliptin treatment and DPP4 deficiency reduce social fear and prevent the onset of comorbid depressive-like behavior. The reduced efficacy of sitagliptin in NPY-deficient mice highlights the role of NPY in mediating these effects. Our findings emphasize the complex interplay between DPP4 and NPY in modulating social fear and suggest that early intervention with sitagliptin could offer significant clinical promise for treating SAD. By alleviating social fear and preventing the onset of comorbid depressive-like behavior, sitagliptin may provide a dual therapeutic benefit for SAD patients. Given its approval for type 2 diabetes mellitus, sitagliptin’s potential repurposing for SAD offers an opportunity for faster clinical application, providing a promising strategy to address both the core symptoms of SAD and the associated mood disorders that often complicate treatment outcomes.

## Supplementary information

Supplemental Information

## Data Availability

The data generated during the current study are available from the corresponding author on request.
