# Supplementary material for: Sitagliptin as a therapeutic approach for social anxiety disorder: the role of DPP4 and NPY in modulating social fear and comorbid depressive-like behavior in mice
Source: Neuropsychopharmacology. 2025 Jun 9;50(11):1724–31. doi: 10.1038/s41386-025-02146-8 (PMC12436601; doi:10.1038/s41386-025-02146-8)
Supplement: Supplementary file 1 — Supplemental Information [file 41386_2025_2146_MOESM1_ESM.pdf]

## Supplementary Materials and Methods

### Animals

DPP4-deficient mice (DPP4−/−; Dpp4tm1Nwa/Orl) were generated on a C57BL/6 background trough targeted deletion of DPP4 exons 1 and 2, as previously described [32], resulting in the inactivation of DPP4 expression. DPP4−/− mice are fertile and do not exhibit developmental or phenotypic abnormalities such as motor deficits or disturbances in eating and drinking [32].

NPY-deficient mice (NPY−/−; 129S-Npytm1Rpa/J) were generated on a C57BL/6 background, as previously described [34], and were generously provided by Prof H. Herzog, Garvan Institute of Medical Research, Sydney, Australia. NPY−/− mice are fertile and do not display developmental abnormalities. Behaviorally, NPY−/− mice show normal learning and memory abilities [34,35], but exhibit increased anxiety-like behavior and reduced locomotor activity in aversive areas such as the center of the open field and the light compartment of the light-dark box. In non-aversive areas, NPY−/− mice show normal locomotor activity [33,34].

## Supplementary Tables

| Mice | Gender | Genotype | Conditioning | Treatment | Animal number |
|---|---|---|---|---|---|
| **CD1** | male | | SFC− | Water | n=7+7 (batch1+2) |
| | | | SFC+ | Water | n=7+7 (batch1+2) |
| | | | | Sitagliptin 50 mg/kg/day | n=8+8 (batch1+2) |
| | | | | Sitagliptin 100 mg/kg/day | n=8+8 (batch1+2) |
| **DPP4** | male | +/+ | SFC− | | n=4 |
| | | | SFC+ | | n=6 |
| | | −/− | SFC− | | n=6 |
| | | | SFC+ | | n=6 |
| | female | +/+ | SFC− | | n=3 |
| | | | SFC+ | | n=2 |
| | | −/− | SFC− | | n=2 |
| | | | SFC+ | | n=3 |
| **NPY** | male | +/+ | SFC− | Water | n=6 |
| | | | SFC+ | Water | n=7 |
| | | | | Sitagliptin 100 mg/kg/day | n=7 |
| | | −/− | SFC− | Water | n=6 |
| | | | SFC+ | Water | n=6 |
| | | | | Sitagliptin 100 mg/kg/day | n=6 |
| | female | +/+ | SFC− | Water | n=6 |
| | | | SFC+ | Water | n=5 |
| | | | | Sitagliptin 100 mg/kg/day | n=5 |
| | | −/− | SFC− | Water | n=6 |
| | | | SFC+ | Water | n=4 |
| | | | | Sitagliptin 100 mg/kg/day | n=4 |

**Table S1. Detailed depiction of the experimental groups**. DPP4, dipeptidyl dipeptidase 4; NPY, neuropeptide Y; +/+, wild-type mice; −/−, homozygous deficient mice; SFC−, unconditioned control mice; SFC+, socially fear conditioned mice. CD1 mice: the behavioral analysis and serum corticosterone (CORT) measurements were performed in both batches, while DPP4 activity and protein levels were assessed in batch 1.

| CD1 mice (Figure S1) | group effect<br>day effect | group x day effect |
|---|---|---|
| Body weight curve (S1a) | F(3,56)=2.658; p=0.057<br>F(13,728)=253.487; p<0.001 | F(39,728)=1.357; p=0.075 |
| Mean body weight (S1b) | F(3,56)=2.658; p=0.057 | |
| Weight gain (S1c) | F(3,56)=0.492; p=0.689 | |
| Fluid intake curve (S1d) | F(3,56)=0.728; p=0.540<br>F(12,672)=9.376; p<0.001 | F(36,672)=0.545; p=0.987 |
| Mean fluid intake (S1e) | F(3,56)=0.928; p=0.433 | |
| Mean sitagliptin consumption 50 mg/kg/day (S1f) | T(15)=1.380; p=0.188 | |
| Mean sitagliptin consumption 100 mg/kg/day (S1f) | T(15)=1.131; p=0.276 | |
| **NPY-deficient mice (Figure S2)** | **group effect<br>sex effect<br>day effect** | **group x sex effect<br>day x group effect<br>group x sex x day effect** |
| Body weight curve (S2a) | F(5,56)=19.535; p<0.001<br>F(1,56)=340.719; p<0.001<br>F(12,672)=193.806; p<0.001 | F(5,56)=11.686; p<0.001<br>F(60,672)=1.204; p=0.147<br>F(60,672)=0.434; p=1.000 |
| Mean body weight (S2b) | F(5,56)=20.092; p<0.001<br>F(1,56)=350.950; p<0.001 | F(5,56)=12.065; p<0.001 |
| Weight gain (S2c) | F(5,56)=1.261; p=0.294<br>F(1,56)=0.012; p=0.913 | F(5,56)=0.478; p=0.791 |
| Fluid intake curve (S2d) | F(5,56)=4.606; p=0.001<br>F(1,56)=345.107; p<0.001<br>F(11,616)=3.250; p<0.001 | F(5,56)=9.245; p<0.001<br>F(55,616)=1.603; p=0.005<br>F(55,616)=1.040; p=0.400 |
| Mean fluid intake (S2e) | F(5,56)=3.817; p=0.005<br>F(1,56)=364.746; p<0.001 | F(5,56)=10.030; p<0.001 |
| Mean sitagliptin consumption (NPY+/+ versus NPY−/− mice) (S2f) | F(1,18)=2.968; p=0.102<br>F(1,18)=20.324; p<0.001 | F(1,18)=0.125; p=0.728 |
| Mean sitagliptin consumption (NPY+/+ mice) (S2f) | T(11)=1.246; p=0.238 | |
| Mean sitagliptin consumption (NPY−/− mice) (S2f) | T(9)=-0.587; p=0.572 | |
| Sitagliptin consumption curve (NPY+/+ versus NPY−/− mice) | F(1,18)=2.968; p=0.102<br>F(1,18)=20.324; p<0.001<br>F(11,198)=0.784; p=0.655 | F(1,18)=0.125; p=0.728<br>F(11,198)=0.661; p=0.774<br>F(11,198)=1.491; p=0.137 |

**Table S2. Statistical details regarding body weight, fluid intake and sitagliptin consumption in the presented data.** NPY, neuropeptide Y; +/+, wild-type mice; −/−, homozygous deficient mice; Weight gain is defined as the difference in body weight between the first and last day of the

experiment. Fluid intake represents the daily intake of water or sitagliptin, measured in ml/g/day. Sitagliptin consumption refers to the amount of sitagliptin consumed per day, expressed in mg/kg/day. CD 1 mice: the mean sitagliptin consumption evaluated each group against the fixed value of 50 or 100 (mg/kg/day) to investigate how consistent was the sitagliptin consumption within each group. NPY-deficient mice: the mean sitagliptin consumption was compared between the two genotypes (NPY+/+ versus NPY−/− mice) to investigate possible genotype-dependent differences. Both genotypes were also evaluated against the fixed value of 100 (mg/kg/day) (NPY+/+ mice; NPY−/− mice) to investigate how consistent was the sitagliptin consumption within each group.

| **DPP4 deficiency (Figure 3)** | **sex effect** | **conditioning x sex effect**<br>**conditioning x genotype x sex effect** |
|---|---|---|
| SFC investigation (3a) | F(1,24)=5.459; p=0.028 | F(1,24)=0.323; p=0.575<br>F(1,24)=0.043; p=0.837 |
| SFC foot shocks (3b) | F(1,13)=2.122; p=0.169 | F(1,13)=0.531; p=0.479 |
| SFC extinction (3c) | F(1,24)=0.979; p=0.332 | F(1,24)=0.002; p=0.962<br>F(1,24)=0.626; p=0.437 |
| EPM time open arms (3d) | F(1,24)=0.650; p=0.428 | F(1,24)=0.383; p=0.542<br>F(1,24)=3.506; p=0.073 |
| EPM closed arm entries (3e) | F(1,24)=0.544; p=0.468 | F(1,24)=2.341; p=0.139<br>F(1,24)=0.177; p=0.678 |
| FST immobility (4f) | F(1,24)=6.046; p=0.022 | F(1,24)=0.077; p=0.784<br>F(1,24)=0.175; p=0.679 |
| **NPY deficiency (Figure 4)** | **sex effects** | **group x sex effects** |
| SFC investigation (4a) | F(1,56)=0.024; p=0.876 | F(5,56)=0.138; p=0.983 |
| SFC foot shocks (4b) | F(1,36)=1.988; p=0.167 | F(3,36)=0.306; p=0.821 |
| SFC extinction (4c) | F(1,56)=0.752; p=0.389 | F(5,56)=0.444; p=0.816 |
| EPM time open arms (4d) | F(1,56)=2.513; p=0.119 | F(5,56)=0.151; p=0.979 |
| EPM closed arm entries (4e) | F(1,56)=1.328; p=0.254 | F(5,56)=0.141; p=0.982 |
| FST immobility (4f) | F(1,56)=3.877; p=0.054 | F(5,56)=0.369; p=0.868 |

**Table S3. Statistical details regarding sex differences in the behavioral data.** DPP4, dipeptidyl dipeptidase 4; NPY, neuropeptide Y; SFC, social fear conditioning; EPM, elevated plus-maze test; FST, forced swim test.

## Supplementary Figures

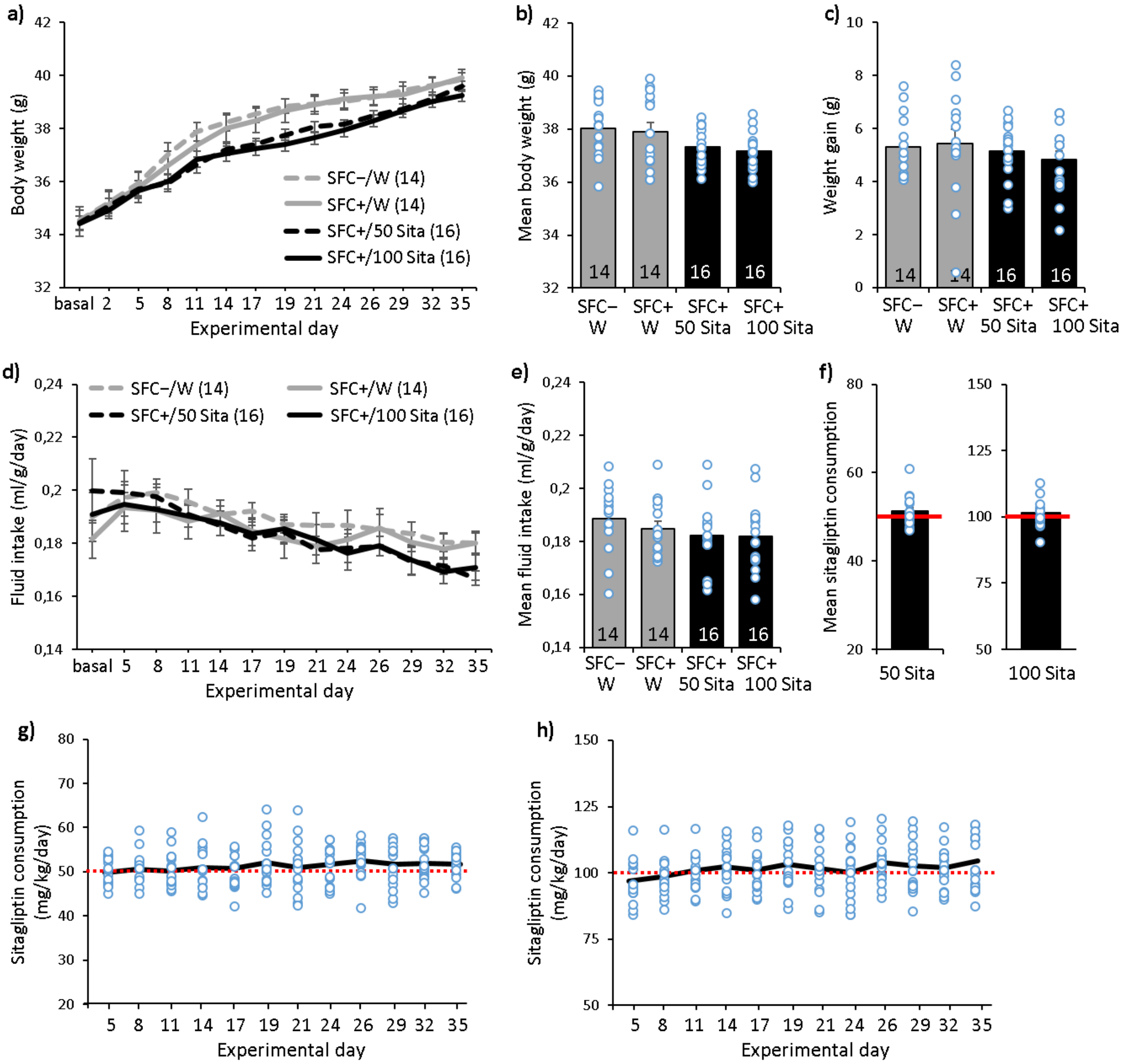


**Figure S1. Body weight, weight gain, fluid intake and sitagliptin consumption in male CD1 mice.** SFC−, unconditioned control mice; SFC+, socially fear conditioned mice. Sitagliptin (Sita) was administered for 4 weeks via drinking water (W) at a daily dose of 50 mg/kg or 100 mg/kg. The treatment was started one day after SFC (on experimental day 2) to avoid potential treatment-induced alterations in social fear memory consolidation and was maintained throughout the experiment. The mice's body weight and drinking volume were assessed every two to three days, and the sitagliptin solution was freshly prepared accordingly. Weight gain (**c**) refers to the difference in body weight between basal and experimental day 35. Fluid intake (**d**) represents the daily intake of water or sitagliptin. There was no difference in body weight (**a,b**), weight gain (**c**) and fluid intake (**d,e**) between the groups. The sitagliptin consumption was consistent within each group when evaluated against the fixed value of 50 (mg/kg/day) (**f,g**) and 100 (mg/kg/day) ($p<0.05$) (**f,h**).

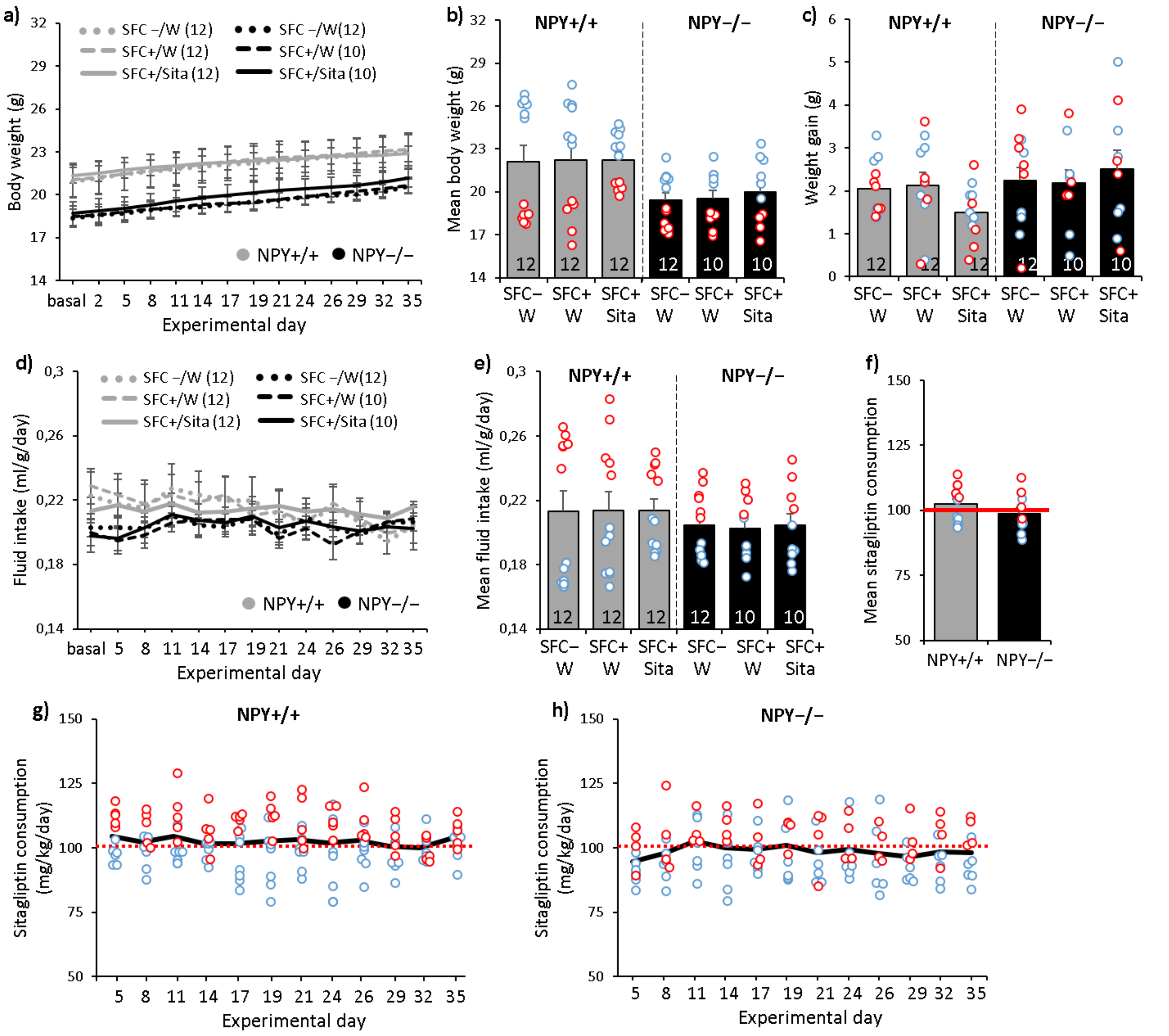


**Figure S2. Body weight, weight gain, fluid intake and sitagliptin consumption in male and female NPY-deficient mice.** NPY, neuropeptide Y; +/+, wild-type mice; −/−, homozygous deficient mice; SFC−, unconditioned control mice; SFC+, socially fear conditioned mice. Sitagliptin (Sita) was administered for 4 weeks via drinking water (W) at a daily dose of 100 mg/kg. The treatment was started one day after SFC (on experimental day 2) to avoid potential treatment-induced alterations in social fear memory consolidation and was maintained throughout the experiment. The mice's body weight and drinking volume were assessed every two to three days, and the sitagliptin solution was freshly prepared accordingly. Weight gain (**c**) refers to the difference in body weight between basal and experimental day 35. Fluid intake (**d**) represents the daily intake of water or sitagliptin. Males showed a higher daily body weight (**a**; from basal to experimental day 35) and mean body weight (**b**) than females in all groups (p<0.001), independent of genotype and treatment. Male NPY+/+ mice were heavier that male NPY−/− mice, independent of treatment (**a**; basal – day 35; **b**; mean body weight); this genotype difference in body weight was observed only in sitagliptin-treated females but not in water-drinking females (**a,b**), mainly because sitaglitpin-treated NPY+/+ females showed from the beginning a generally higher body weight than water-drinking NPY+/+ females (**a,b**). Both males and females showed a significant

body weight gain (**a**; basal – day 35), independent of genotype and treatment; the overall weight gain from basal to day 35 was not influenced by genotype or treatment (**c**). Females showed a higher daily fluid intake (**d**; from basal to experimental day 35) and mean fluid intake (**e**) than males in all groups ($p<0.05$), independent of genotype and treatment. Female NPY+/+ mice showed a higher water intake than female NPY−/− mice; this genotype difference in fluid intake was not observed in sitagliptin-treated females (**d,e**). In males, there was no genotype or treatment effect in fluid intake (**d,e**). In general, females consumed more sitagliptin than males, independent of genotype (**g**; NPY+/+ during day 5, 11, 17, 19, 21, 24; **h**; NPY−/− during day 5, 14, 2, 32, 35). Both female NPY+/+ mice ($p=0.002$) and female NPY−/− mice ($p=0.012$) showed a higher mean sitagliptin consumption than respective males (**f**), but there was no genotype difference in sitaglitpin consumption either in females or males. The sitagliptin consumption was consistent within each group when evaluated against the fixed value of 100 (mg/kg/day; males and females combined; $p<0.05$) (**f,g,h**).

**Quantification of serum neuropeptide Y (NPY) protein levels in socially fear conditioned male CD1 mice in a pilot analysis**

To verify whether sitagliptin increases NPY levels, CD1 mice were rapidly killed after 39 days of sitagliptin treatment (50 mg/kg/day via drinking water). Trunk blood was collected via cardiac puncture and left to coagulate. After centrifugation (4°C, 4000 rpm, 10 min), the serum was extracted and stored at –80°C. NPY protein levels were quantified in 8 µL serum duplicates using a competitive NPY Extraction Free EIA Kit (EKE-049-03, Phoenix Europe GmbH, Karlsruhe, Germany), based on a standard curve ranging from 0.1 to 40 ng/mL, within one assay with an intra-assay coefficient of variation of 3%.

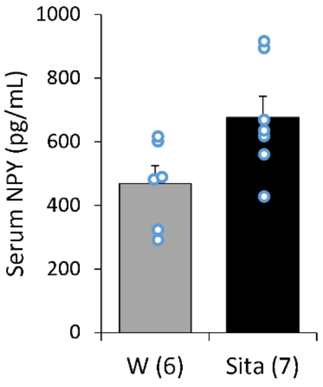


**Figure S3. Sitagliptin increases serum NPY levels in socially fear conditioned male CD1 mice.** NPY, neuropeptide Y. All mice were socially fear conditioned and sitagliptin (Sita) treatment was started one day later to avoid potential treatment-induced alterations in social fear memory consolidation. Sitagliptin was administered for 4 weeks via drinking water (W) at a daily dose of 50 mg/kg. Data represent means + SEM and numbers on the bars indicate group sizes. * $p<0.05$
